# Toward Intelligent Sensing Systems: Non-Equilibrium Materials as Platforms for AI-Enabled Autonomous Discovery

**DOI:** 10.3390/s26103036

**Published:** 2026-05-12

**Authors:** Ashutosh Tiwari, Gitanjali Mishra, Jagdish Narayan

**Affiliations:** 1 Department of Materials Science and Engineering, University of Utah, Salt Lake City, UT 84112, USA; 2 Department of Materials Science and Engineering, North Carolina State University, Raleigh, NC 27695, USA

**Keywords:** non-equilibrium materials, defects and disorder, in-sensor computing, autonomous discovery, artificial intelligence, machine learning, adaptive materials

## Abstract

Conventional sensing systems rely on sequential architectures in which signal acquisition, processing, and decision-making are physically and functionally separated. This paradigm imposes limitations in latency, energy efficiency, and adaptability, particularly in data-intensive and dynamic environments. In this perspective, we discuss an emerging framework for intelligent sensing systems in which these functions are increasingly integrated through the intrinsic properties of functional materials. Non-equilibrium materials exhibit nonlinearity, memory, temporal dynamics, and adaptive responses that enable in-sensor information transformation. When coupled with artificial intelligence, these material capabilities support sensing platforms capable of encoding, processing, and interpreting information at or near the point of measurement. We examine key material platforms, architectural strategies, and opportunities for closed-loop autonomous discovery, while also highlighting challenges related to variability, scalability, and system integration. This convergence of materials science and intelligent systems points toward sensing technologies that move beyond passive measurement toward adaptive, low-latency, and energy-efficient operation.

## 1. Introduction: From Passive Sensors to Intelligent Systems

The rapid evolution of sensing technologies has enabled unprecedented capabilities in monitoring physical, chemical, and biological environments. Conventional sensing systems, however, remain fundamentally constrained by a sequential architecture in which sensing, data acquisition, and computation are treated as distinct stages. Within this framework, materials function primarily as transducers that convert external stimuli into electrical signals, which are then processed through external electronics or computational units. While this paradigm has supported the development of highly sensitive and selective sensors, it introduces inherent limitations in latency, energy efficiency, and scalability, particularly in applications requiring real-time response and distributed intelligence [1,2,3,4,5].

Recent advances in materials science, device physics, and artificial intelligence are motivating a shift toward more integrated approaches in which sensing and information processing are more tightly coupled. Rather than serving solely as passive elements, functional materials are increasingly being explored as active components whose intrinsic physical responses can contribute directly to how information is represented and manipulated. This shift is particularly relevant in the context of edge computing and autonomous systems, where constraints on power, bandwidth, and response time necessitate more efficient data handling. In such settings, localized information processing at or near the point of sensing can significantly enhance system performance. Material platforms exhibiting complex, stimulus-dependent responses offer a pathway toward this capability, as their behavior can encode input signals in nonlinear and history-dependent ways, reducing reliance on downstream processing [3,4,6].

**Figure 1:**
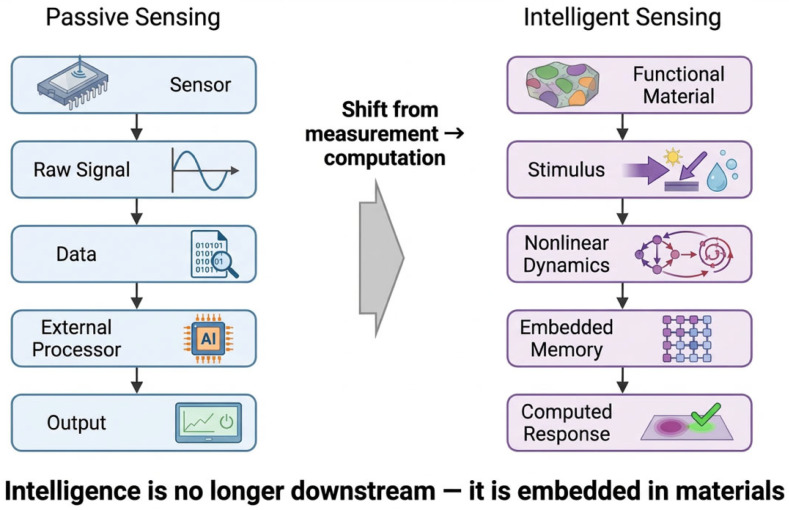
Paradigm shift from conventional sensing to intelligent material-based information processing. Traditional sensing architectures operate through a sequential pipeline, where sensors transduce external stimuli into electrical signals for downstream processing. In contrast, emerging intelligent sensing systems leverage intrinsic material properties—such as nonlinearity, memory, and dynamic response—to enable in-situ information transformation. Functional materials thus move beyond passive transduction to actively encode and process information, allowing computation to occur within the material itself. This transition represents a fundamental shift from measurement to embedded computation.

In this perspective, we examine how non-equilibrium material systems—characterized by features such as defects, disorder, and metastability—can serve as platforms for next-generation sensing architectures (Figure 1). We discuss how these material characteristics give rise to rich dynamical behavior and how such behavior can be leveraged to enable new modes of operation beyond conventional sensing. By connecting advances in materials synthesis with emerging concepts in intelligent system design, this work outlines a pathway toward sensing technologies in which material properties play a central and active role in shaping functionality [6,7,8].

## 2. Non-Equilibrium Materials as Platforms for Intelligent Functionality

The emergence of intelligent sensing systems is closely linked to advances in materials that exhibit complex, dynamic, and tunable responses to external stimuli. In contrast to equilibrium materials—where properties are largely governed by thermodynamic minima—non-equilibrium materials provide access to metastable phases, defect configurations, and interfacial states that enable rich functional behavior. These materials are particularly attractive for next-generation sensing platforms, as they naturally exhibit nonlinearity, memory, and sensitivity to perturbations, which are essential for embedding intelligence at the material level. Through non-equilibrium synthesis routes, it becomes possible to engineer responses that extend beyond simple transduction, enabling functionalities that bridge sensing and computation. More fundamentally, such materials can not only detect external stimuli but also encode, transform, and process information intrinsically, forming the physical basis for intelligent functionality.

### 2.1. Nonlinearity as a Functional Resource

Nonlinearity is a fundamental requirement for transforming input signals into rich, high-dimensional representations and plays a central role in enabling intelligent sensing. While conventional sensing systems favor linear responses for ease of calibration and interpretation, such responses inherently limit the ability to encode complex relationships between inputs and outputs. In contrast, nonlinear material responses enable amplification, thresholding, and signal mixing, which are essential for advanced sensing and information processing [9,10,11]. In non-equilibrium materials, nonlinearity arises naturally from mechanisms such as carrier trapping, ionic migration, phase transitions, and interfacial effects. These processes produce input–output relationships that depend strongly on stimulus intensity, frequency, and history. For example, memristive systems exhibit nonlinear switching in current–voltage characteristics, while phase-change materials display abrupt transitions between distinct states. Such behavior enables materials to act as intrinsic signal processors, performing functions including threshold detection, gain control, and nonlinear filtering directly at the sensing level [9,10,12].

Nonlinear responses can enhance sensitivity by amplifying small variations in input signals. Near critical points or transition thresholds, small perturbations can produce large changes in response, enabling high-resolution detection. At the same time, saturation and bounded responses help suppress noise and maintain stable operation, providing a balance between sensitivity and robustness [11,13].

From a computational perspective, nonlinearity enables the mapping of low-dimensional inputs into higher-dimensional feature spaces, a capability central to frameworks such as reservoir computing. In this context, temporal patterns are processed through the intrinsic dynamics of the material system, allowing computation to emerge directly from physical response. Nonlinear materials therefore provide a natural substrate for embedding computation within sensing, reducing reliance on external processing units. Nonlinearity should thus be viewed not as a limitation, but as a functional resource that enables sensing systems to perform sophisticated signal transformations. Leveraging nonlinear responses enables the design of physics-driven sensing platforms capable of real-time processing and adaptive operation in complex environments [9,14,15].

### 2.2. Defects and Disorder as Drivers of Functional Dynamics

A defining characteristic of non-equilibrium materials is the presence of defects, disorder, and complex interfaces, which play a central role in determining functional behavior. While traditionally treated as imperfections to be minimized, defects in many materials act as active sites that govern electronic transport, chemical reactivity, and sensitivity to external stimuli. In the context of intelligent sensing, these features provide a natural basis for nonlinear and adaptive responses. Point defects such as vacancies, interstitials, and substitutional dopants introduce localized energy states within the band structure, enabling nonlinear conduction mechanisms including trap-assisted transport, hopping conduction, and field-dependent mobility. Extended defects and interfaces further contribute to spatial inhomogeneity, creating networks of conduction pathways that respond dynamically to external inputs. Such behavior is particularly relevant in sensing environments where signals are weak, noisy, or time-dependent [7,16,17,18,19,20,21].

Transparent oxide systems, including ZnO and related materials, exemplify the functional role of defects and disorder. Oxygen vacancies, dopant distributions, and surface states strongly influence electrical conductivity and surface adsorption processes, making these materials highly sensitive to chemical species. Their response is inherently nonlinear and depends on factors such as gas concentration, temperature, and prior exposure history. This combination of sensitivity and nonlinear response enables functions analogous to signal processing, including amplification, filtering, and pattern recognition [16,17,21].

Transparent oxide systems, including ZnO and related materials, exemplify the functional role of defects and disorder. Oxygen vacancies, dopant distributions, and surface states strongly influence electrical conductivity and surface adsorption processes, making these materials highly sensitive to chemical species. Their response is inherently nonlinear and depends on factors such as gas concentration, temperature, and prior exposure history. Nonlinear, history-dependent material responses can be interpreted within the framework of reservoir computing, where intrinsic dynamics map input signals into high-dimensional temporal representations suitable for downstream inference. Although such responses do not directly perform decision-making, they enable signal transformation and feature extraction within the material, supporting functions analogous to amplification, filtering, and enabling selective discrimination [16,17,21].

**Figure 2:**
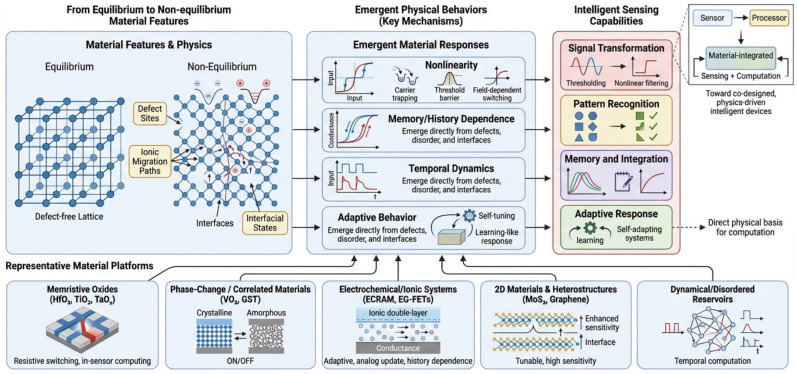
Non-equilibrium material features (defects, disorder, interfaces, metastability) give rise to nonlinear, hysteretic, and time-dependent physical behaviors. These dynamics enable intrinsic information encoding and support functionalities such as signal transformation, memory, pattern recognition, and adaptive response, linking material properties directly to intelligent sensing capabilities.

Interfaces—whether between different materials or between a material and its environment—introduce additional degrees of freedom. Charge transfer, band alignment, and interfacial states can produce history-dependent behavior and hysteresis, enhancing the ability to encode and process information. Rather than being undesirable, such characteristics can be harnessed for material-level computation, where the response carries information about both current and past inputs [18,19,20]. Taken together, defects, disorder, and interfaces transform materials from passive sensing elements into active systems capable of complex signal transformation. These intrinsic properties provide the physical basis for integrating sensing and computation, enabling intelligent sensing architectures built directly upon material functionality. These relationships between non-equilibrium material features and emergent functional behavior are schematically illustrated in Figure 2.

### 2.3. Memory Effects and Temporal Dynamics

Memory is a defining characteristic of intelligent systems, enabling responses that depend not only on current inputs but also on past states. In material systems, memory arises from coupled processes such as charge trapping, ionic migration, structural relaxation, and phase coexistence, which introduce temporal correlations into system behavior. In many non-equilibrium materials, memory manifests as hysteresis, relaxation, and time-dependent conductance changes. Memristive systems, for example, exhibit conductance states that evolve gradually in response to applied stimuli and persist after the stimulus is removed. Phase-change materials retain information through metastable structural configurations, while electrochemical systems store information through ionic redistribution. These mechanisms provide a basis for analog memory, where system states encode a continuous history of prior inputs [14,22,23].

Temporal dynamics associated with these processes often span multiple time scales, from fast electronic responses to slower ionic or structural evolution. This distribution of time constants enables fading memory, in which the influence of past inputs decays over time while maintaining responsiveness to new inputs. Such behavior is essential for processing time-dependent signals, allowing integration over finite temporal windows [14,22].

The interplay between memory and dynamics enables materials to perform temporal signal processing functions such as integration, delay, and pattern recognition. Within dynamical systems frameworks, these properties allow materials to act as reservoirs that transform input signals into high-dimensional temporal representations, a principle widely explored in physical reservoir computing. Beyond computation, memory effects also contribute to adaptive behavior in sensing systems. By retaining information about prior stimuli or environmental conditions, materials can adjust their response characteristics over time, enabling learning-like functionality. Memory should therefore be viewed not as static storage, but as an active component of material dynamics, enabling continuous and context-aware processing [14,15,22,23,24].

### 2.4. Representative Material Platforms for Intelligent Sensing

Recent advances in non-equilibrium materials have enabled a diverse set of platforms that intrinsically combine sensing, memory, and computation. Rather than serving as passive transducers, these systems exhibit nonlinear, history-dependent, and time-evolving responses that can be directly harnessed for intelligent sensing.

Memristive oxide systems, including HfO_2_-, TiO_2_-, and TaO_X_-based materials, are among the most extensively studied platforms for in-sensor computation. Their resistive switching behavior, governed by ionic migration and defect redistribution, gives rise to nonlinear conductance modulation and nonvolatile memory. These properties enable temporal integration of input signals and analog state retention, allowing memristive devices to function as both sensors and computational elements. Such systems have been widely explored for neuromorphic sensing and edge intelligence [7,12,13,25]. Complementary to these, phase-change and correlated materials such as Ge_2_Sb_2_Te_5_ (GST) and VO_2_ exhibit abrupt and hysteretic transitions between distinct electronic or structural phases driven by thermal, electrical, or optical stimuli. The coexistence of multiple metastable states enables multilevel memory and threshold-based switching, while dynamic phase evolution supports time-dependent signal processing [10,26,27,28].

Electrochemical and ionic systems, including electrolyte-gated transistors and electrochemical random-access memory (ECRAM), offer a direct interface between environmental stimuli and material response. In these systems, ionic motion modulates electronic conductance in a continuous and analog manner, enabling gradual, history-dependent updates of the system state. This coupling between ionic and electronic transport provides a natural mechanism for adaptive sensing, particularly in chemical and biological environments [23,29]. Two-dimensional materials and van der Waals heterostructures, such as MoS_2_, graphene, and layered hybrid systems, provide highly tunable platforms for intelligent sensing. Their large surface-to-volume ratio enhances sensitivity to external stimuli, while defect engineering and interfacial coupling introduce nonlinear and memory effects. By combining multiple layers with distinct properties, these systems can support multifunctional sensing and integrated signal processing within compact architectures [19,24,30,31,32].

To provide a concrete illustration of how these concepts translate into practical systems, a representative example is provided by oxide-based gas sensing systems such as ZnO, where time-dependent conductivity changes arising from adsorption and desorption of gas molecules can be treated as temporal signals. Exposure to different gases or concentration profiles produces distinct nonlinear and history-dependent response trajectories due to variations in surface reactions and defect states. These time-evolving responses can be interpreted as a transformation of input stimuli into higher-dimensional representations through intrinsic material dynamics. A simple output mapping, such as a trained linear classifier or regression-based estimator, can then be used to distinguish between different gases or operating conditions. This illustrates how material response can perform feature transformation within the sensing layer, consistent with reservoir computing principles.

More generally, dynamical and disordered systems have emerged as promising substrates for reservoir computing. In such platforms, intrinsic temporal dynamics and nonlinear responses transform input signals into high-dimensional representations, enabling real-time processing of time-dependent data without explicit programming. This perspective highlights the broader potential of non-equilibrium materials as computational media, where functionality emerges directly from underlying physical processes [15,24,33,34,35,36]. Collectively, these platforms demonstrate that intelligent sensing can arise from intrinsic material dynamics.

From a design perspective, effective implementation of intelligent sensing systems requires tuning key material properties such as nonlinearity, relaxation dynamics, and memory retention to match the temporal characteristics of the input signals. Operating conditions, including biasing schemes and stimulus modes, further determine how these intrinsic dynamics are activated and utilized for sensing, signal transformation, and temporal encoding. The critical next step is to translate these properties into device architectures and system-level implementations, where sensing and computation are co-designed rather than sequentially separated.

## 3. From Material Dynamics to Intelligent Sensing Architectures

**Figure 3:**
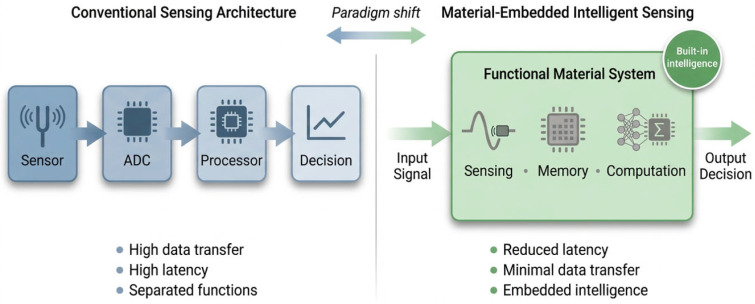
Schematic comparison between conventional sensing architectures and material-embedded intelligent sensing. Traditional systems rely on sequential processing steps (sensor, ADC, processor, decision), leading to high data transfer and latency. In contrast, functional material systems integrate sensing, memory, and computation within the material itself, enabling reduced latency, minimal data transfer, and embedded intelligence.

The transition from conventional sensing to intelligent sensing systems is driven not only by advances in material properties, but also by how these properties are organized within system-level architectures. Traditional sensing platforms are structured as linear pipelines in which signal acquisition, transmission, and processing occur in distinct stages. While effective for many applications, such architectures separate physical interaction from information processing, resulting in increased latency, higher energy consumption, and limited adaptability. Emerging architectures address these limitations by more tightly integrating material response with system functionality, enabling signal transformation to begin at the point of interaction with the environment. In these systems, sensing and processing are no longer strictly sequential, but occur in a coupled and often concurrent manner (Figure 3).

### 3.1. From Sequential Pipelines to Integrated Systems

Conventional sensor architectures rely on well-defined stages—detection, signal conditioning, data acquisition, and external computation—each typically optimized independently, with materials confined to signal transduction. While this modular design offers flexibility, it introduces bottlenecks associated with data transfer and processing overhead. In contrast, integrated sensing architectures redistribute functionality across the system, allowing portions of signal transformation to occur within the sensing layer itself. This approach does not eliminate external computation, but reduces its burden by embedding preliminary processing within the material response. As a result, systems can achieve improved efficiency, lower latency, and reduced power consumption, particularly in applications requiring rapid decision-making or operation under resource constraints. Furthermore, this integration enables more compact and scalable designs by relaxing the rigid separation between sensing and processing units [37,38].

### 3.2. In-Sensor and Edge-Level Processing

A key development in this direction is the emergence of in-sensor processing, where aspects of signal interpretation are performed locally within or near the sensing material. This approach aligns with edge computing paradigms, in which data is processed close to its source to minimize latency and bandwidth requirements. In-sensor processing ranges from basic operations such as filtering and feature extraction to more complex transformations that encode nonlinear and temporal characteristics of input signals. Materials exhibiting state-dependent or time-evolving responses are particularly well suited for such roles, as they naturally capture signal history and context. By performing these operations locally, sensing systems can reduce data transmission demands, improve energy efficiency, and enable operation in distributed and resource-constrained environments such as wearable systems and autonomous platforms. Importantly, in-sensor processing complements rather than replaces digital computation, forming hybrid systems in which physical and algorithmic processing are co-optimized. In practical implementations, such approaches are realized through device platforms including memristive arrays, electrolyte-gated transistors, and hybrid sensor–processor architectures, where material dynamics are directly interfaced with electronic readout and control circuitry [39,40,41,42].

### 3.3. Distributed and Dynamical Sensing Frameworks

Beyond localized processing, intelligent sensing architectures increasingly adopt distributed and dynamical frameworks in which information is represented across multiple interacting elements. In these systems, the overall response emerges from the collective behavior of material and device networks rather than from a single sensing unit. Materials exhibiting spatial and temporal heterogeneity—arising from variations in structure, defects, or environmental exposure—naturally support such distributed representations. When harnessed effectively, this diversity enhances the system’s ability to encode complex input signals, as different regions provide distinct projections of the same stimulus. Architecturally, this leads to sensing platforms that function as distributed dynamical networks, where system evolution encodes both present and past inputs. Such frameworks align closely with emerging computational paradigms based on dynamical systems, including reservoir-like approaches and physics-based computation [33,34,35,36].

### 3.4. Toward Closed-Loop and Adaptive Systems

The integration of material response, sensing, and processing further enables closed-loop architectures in which system behavior is continuously adjusted based on incoming data. Feedback mechanisms—implemented through electronic control, environmental modulation, or adaptive material states—allow real-time refinement of system performance. This capability is particularly important in applications such as autonomous discovery, environmental monitoring, and adaptive control, where operating conditions evolve dynamically. By incorporating feedback across multiple levels, these systems can adjust sensitivity, selectivity, and operational parameters without external intervention. While fully autonomous sensing platforms remain an active area of research, ongoing advances in materials, device integration, and data-driven methods point toward systems that are not only responsive but capable of continuous self-optimization [43,44,45,46].

## 4. AI-Enabled Autonomous Sensing and Discovery

The convergence of advanced sensing platforms with artificial intelligence is enabling a transition toward autonomous, closed-loop systems capable of real-time decision-making and adaptive operation. In these systems, sensing, data interpretation, and actuation form a unified cycle rather than distinct stages. When combined with materials exhibiting intrinsic nonlinearity and temporal dynamics, this integration enables tight coupling between sensing and computation across multiple scales.

### 4.1. Closed-Loop Sensing and Decision Systems

Closed-loop sensing systems operate through continuous cycles of measurement, analysis, and response, in which outputs influence subsequent inputs through feedback. Unlike open-loop systems that rely on external processing, these architectures incorporate AI-driven decision-making directly within the sensing workflow, forming iterative loops of sensing → inference → actuation → updated sensing. Sensor outputs are processed in real time using machine learning models to extract features and generate actionable insights, which guide actuation by adjusting system parameters or environmental conditions. The effectiveness of such systems depends on the latency, fidelity, and adaptability of the sensing layer. Materials capable of in-sensor processing and dynamic response enhance performance by enabling local computation and reducing data transfer. For example, nonlinear material responses can support feature extraction, while memory effects retain information about recent inputs, improving operation in time-dependent environments. These capabilities are particularly relevant in applications such as environmental control, industrial optimization, and biomedical monitoring, where conditions evolve continuously and require rapid adaptation [45,46].

### 4.2. Autonomous Experimentation and Self-Driving Laboratories

Extending closed-loop operation to scientific discovery, autonomous experimentation enables systems that iteratively plan, execute, and analyze experiments with minimal human intervention. In such frameworks, sensor data are continuously integrated with AI-driven models that determine subsequent experimental conditions. In materials science, this approach underpins self-driving laboratories, where synthesis, characterization, and analysis are combined within automated workflows. These systems efficiently explore high-dimensional parameter spaces—including composition, temperature, and processing conditions—using methods such as Bayesian optimization and reinforcement learning to identify optimal material states. Advanced sensing materials further enhance these workflows by providing rich datasets that capture nonlinear and time-dependent behavior, improving model accuracy and decision-making. Additionally, in situ and real-time sensing enables dynamic adjustment of experimental conditions, which is particularly important in processes such as thin-film growth where small parameter variations strongly influence material properties. Autonomous experimentation thus represents a shift toward active, data-driven exploration in which sensing directly informs and accelerates discovery [47,48,49,50,51].

### 4.3. Adaptive and Self-Optimizing Material Systems

A natural extension of closed-loop sensing and autonomous experimentation is the development of adaptive material systems in which functional response evolves under external stimuli and feedback. Unlike conventional materials with fixed properties, these systems possess internal degrees of freedom—such as defect configurations, charge states, ionic distributions, or magnetic ordering—that can be dynamically modified. Mechanisms including defect migration, adsorption–desorption, phase transitions, and interfacial charge redistribution enable reversible or persistent changes in material behavior, allowing the system to retain information about prior inputs. When coupled with AI-driven control, these materials participate in feedback loops where material response informs learning algorithms that adjust external conditions to achieve desired outcomes. Over successive iterations, the system converges toward optimized behavior at both material and system levels. This co-evolution of material and algorithm establishes a paradigm in which learning spans physical and computational domains, enabling sensing platforms that are not only responsive but capable of continuous self-optimization [45,52].

## 5. Materials Platforms Across Sensing Modalities

**Figure 4:**
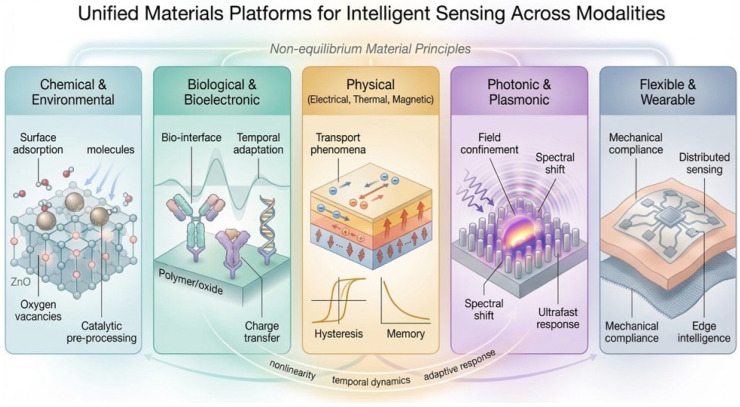
Non-equilibrium material principles enable diverse sensing functionalities in chemical, biological, physical, photonic, and flexible systems. Across these modalities, intrinsic properties such as nonlinearity, temporal dynamics, and adaptive response provide a common foundation for integrating sensing, memory, and computation within material platforms.

While the preceding sections establish general principles linking non-equilibrium materials to intelligent sensing, it is essential to examine how these concepts manifest across different sensing modalities. Chemical, biological, physical, and optical systems are often treated as distinct domains; however, from a materials perspective, they share common foundations rooted in nonlinearity, temporal dynamics, and sensitivity to external perturbations (Figure 4). These shared characteristics provide a unifying framework in which sensing platforms can be viewed not only as transducers but as functional substrates for information processing. This cross-domain perspective highlights the generality of material-driven intelligence and suggests that diverse sensing technologies can be interpreted within a common physical and computational framework [3,6].

### 5.1. Chemical and Environmental Sensing

Chemical and environmental sensing based on semiconducting metal oxides remains one of the most mature and widely deployed platforms. Materials such as ZnO, SnO_2_, TiO_2_, WO_3_, and In_2_O_3_ exhibit strong sensitivity to gaseous species through surface adsorption processes that modulate charge carrier concentration and transport properties. Oxygen vacancies, dopant distributions, and surface states govern these interactions, enabling high sensitivity to changes in ambient conditions [16,17,21,31,53].

The response of these systems is inherently nonlinear and history-dependent, with conductivity influenced not only by instantaneous gas concentration but also by prior exposure and environmental conditions. This temporal behavior enables encoding of evolving chemical environments rather than simple instantaneous detection. When implemented in sensor arrays with diverse response characteristics, such systems support pattern recognition and selective detection with reduced reliance on external computation. Catalytic functionality further enhances selectivity by promoting specific chemical interactions, effectively embedding chemical pre-processing within the material. Oxide-based systems thus provide a platform for analog signal transformation, where material responses capture complex input–output relationships and contribute to integrated sensing and computation [31,53,54,55].

### 5.2. Biological and Wearable Sensing Interfaces

Biological, bioelectronic, and wearable sensing systems operate at the interface between materials and complex, dynamic environments such as the human body. These platforms must detect subtle biochemical and physiological signals—including protein binding, ion concentration changes, mechanical deformation, and cellular activity—under conditions of low signal amplitude, variability, and noise. Materials used in these systems, including functionalized oxides, polymers, biomolecular interfaces, two-dimensional materials, and hybrid nanostructures, exhibit responses governed by adsorption, diffusion, interfacial charge transfer, and strain-dependent effects.

As a result, sensor outputs reflect not only the presence of stimuli but also their temporal evolution, providing richer information than static measurements. This dynamic behavior supports adaptive sensing, where repeated exposure or mechanical interaction modifies response characteristics, effectively tuning sensitivity and selectivity. The distributed nature of wearable systems further enables continuous, spatially resolved monitoring, generating data streams that capture both temporal and spatial variations [56,57,58]. When combined with edge-level processing and AI-driven analysis, these platforms enable real-time interpretation of complex biological data, supporting applications in diagnostics, wearable monitoring, and responsive therapeutic systems. By integrating sensing, processing, and feedback, bioelectronic and wearable systems illustrate how material dynamics enable context-aware and adaptive operation [56,57].

### 5.3. Physical Sensing: Electrical, Thermal, and Magnetic Systems

Physical sensing platforms based on electrical, thermal, and magnetic properties provide a complementary pathway for material-enabled functionality. These systems rely on transport phenomena—including charge conduction, heat flow, and spin dynamics—to detect changes in temperature, pressure, magnetic field, or mechanical stress. In electrically active materials, variations in conductivity or impedance arise from changes in carrier concentration, scattering mechanisms, or structural configuration, while thermal and magnetic sensors exploit temperature-dependent transport and spin-dependent interactions [59,60,61,62].

In many cases, these responses are coupled with memory and hysteresis, particularly in materials with complex microstructures or magnetic ordering. Spin-dependent materials, including diluted magnetic semiconductors and magnetoresistive systems, demonstrate how spin transport introduces additional functionality, enabling simultaneous sensing of electrical and magnetic signals. The presence of hysteresis and relaxation effects allows these materials to retain information about prior states, effectively combining sensing and memory within a single platform. Such multifunctional behavior enables integration of multiple inputs and temporal encoding, extending functionality beyond measurement toward dynamic signal processing [59,60].

### 5.4. Photonic and Plasmonic Sensing Platforms

Photonic and plasmonic sensing platforms leverage light–matter interactions to achieve high sensitivity, rapid response, and label-free detection across chemical and biological domains. Variations in refractive index, absorption, or scattering induced by external stimuli are transduced into optical signals, while nanophotonic and plasmonic structures provide strong field confinement that enhances surface sensitivity [62,63,64,65].

Beyond detection, these platforms enable wave-based information processing through parallelism, interference, and phase modulation. Interference patterns and spectral shifts encode multidimensional information about the environment, allowing simultaneous detection and feature extraction. The ultrafast dynamics of photonic systems make them well suited for real-time sensing in rapidly changing conditions. When combined with tunable or adaptive materials, these platforms support reconfigurable sensing behavior, extending functionality into regimes where detection and information transformation are intrinsically coupled [63,64].

Across these diverse platforms, a common theme emerges: material properties are not merely enabling detection but are increasingly shaping how information is encoded, transformed, and interpreted. This convergence of sensing and computation underscores the versatility of non-equilibrium materials as building blocks for intelligent systems. At the same time, the features that enable this functionality—nonlinearity, variability, and dynamic behavior—introduce challenges in reproducibility, scalability, and system integration, motivating the need for coordinated advances across materials, devices, and system architectures.

## 6. Challenges in Integrating Materials, Sensing, and Intelligence

**Figure 5:**
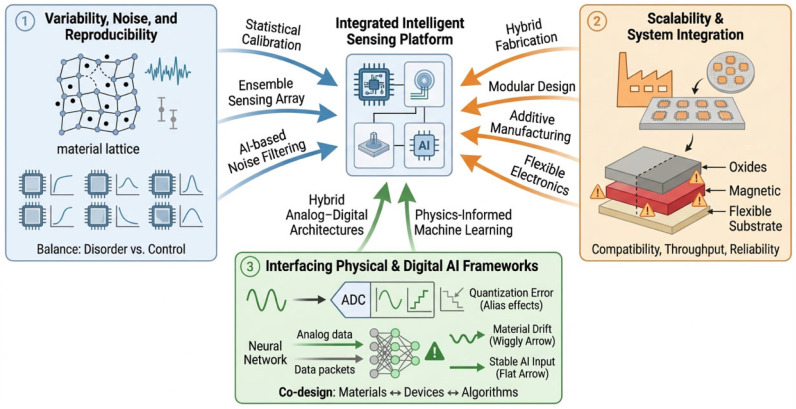
Key challenges and integration pathways for intelligent sensing systems. Variability and noise in material systems necessitate statistical calibration, ensemble sensing, and AI-based filtering. Scalability requires modular design, hybrid fabrication, and compatibility with existing platforms. Bridging physical sensing and digital AI frameworks involves hybrid analog–digital architectures and co-design across materials, devices, and algorithms to enable robust and reliable operation.

The integration of non-equilibrium materials with intelligent sensing architectures presents significant opportunities, but also introduces fundamental challenges that must be addressed for practical implementation (Figure 5). These challenges arise from intrinsic material complexity, constraints in scalable device fabrication, and the need to bridge fundamentally different paradigms of physical and digital information processing. Addressing these issues is essential for translating material-level functionalities into robust and deployable intelligent sensing systems.

### 6.1. Variability, Noise, and Reproducibility

Non-equilibrium materials inherently exhibit variability and disorder arising from fluctuations in growth conditions, defect distributions, and interfacial structures. While such variability can enhance functional richness—enabling nonlinear and adaptive responses—it also poses challenges for reproducibility and reliability. Devices fabricated under nominally identical conditions may exhibit different response characteristics, complicating calibration and standardization.

Noise is a critical factor in systems where sensing relies on subtle changes in electrical, optical, or magnetic signals. Thermal fluctuations, stochastic defect dynamics, and environmental perturbations can introduce variations that obscure meaningful information. In conventional sensing platforms, noise is typically minimized through material purification and signal averaging; however, in intelligent sensing systems, variability and noise can also contribute constructively by increasing the diversity of system responses [66,67,68,69].

The central challenge is therefore not the elimination of variability, but its control and effective utilization. Strategies such as statistical calibration, ensemble sensing using heterogeneous device arrays, and data-driven filtering can mitigate adverse effects while preserving functional advantages. Advances in synthesis control and in situ monitoring will further be required to improve reproducibility. Balancing disorder with control remains a defining issue in the development of material-based intelligent sensing systems [66,68,69].

### 6.2. Scalability and System Integration

Translating material-level functionality into practical sensing systems requires scalable fabrication and seamless integration with device architectures. Although non-equilibrium synthesis techniques enable access to unique material states, they are often associated with limited throughput, complex processing conditions, and sensitivity to environmental parameters, which constrain large-scale deployment.

Integration challenges become more pronounced when combining diverse material systems—such as oxides, magnetic materials, photonic structures, and flexible substrates—within a single platform. Issues related to compatibility, interfacial stability, and thermal mismatch can degrade device performance and long-term reliability. In addition, integrating sensing elements with near-sensor or on-chip processing units requires careful co-design to ensure efficient signal transfer and minimal degradation.

Addressing these challenges will require hybrid fabrication approaches that combine the precision of advanced synthesis with the scalability of industrial manufacturing. Modular system design, in which sensing, processing, and actuation components are co-optimized, offers a promising pathway. Continued advances in additive manufacturing, flexible electronics, and heterogeneous integration are likely to play a key role in enabling scalable intelligent sensing architectures [70].

### 6.3. Interfacing Physical Systems with Digital AI Frameworks

A central challenge in intelligent sensing is bridging the gap between continuous, analog material responses and discrete, digital AI frameworks. Material-based systems often produce signals that are nonlinear, time-dependent, and high-dimensional, whereas most AI algorithms operate on structured digital data. Efficiently translating between these domains without loss of critical information remains a major challenge.

Analog-to-digital conversion introduces quantization errors, latency, and energy overhead, potentially offsetting the advantages of in-sensor processing. Moreover, many AI models assume stable input distributions, while material-based systems may exhibit drift and variability over time. This mismatch complicates model training, calibration, and long-term deployment, particularly in dynamic environments.

Emerging approaches seek to address these limitations through hybrid analog–digital architectures, where portions of computation are performed directly within the physical domain. Physics-informed machine learning and adaptive algorithms can further accommodate variability by incorporating knowledge of underlying material behavior into the learning process. Effective integration will ultimately require co-design across materials, devices, and algorithms, ensuring that sensing platforms and AI frameworks operate synergistically [71,72,73].

Taken together, these challenges highlight that the development of intelligent sensing systems requires coordinated advances across materials, devices, and computational frameworks. While significant progress has been made, key issues remain in achieving reliable, scalable, and adaptive operation under real-world conditions. Addressing these challenges will be critical for transitioning from proof-of-concept demonstrations to robust and widely deployable intelligent sensing technologies.

## 7. Outlook

The convergence of materials science, sensing technologies, and artificial intelligence is redefining how information is acquired, processed, and acted upon. Non-equilibrium materials—characterized by intrinsic nonlinearity, memory, temporal dynamics, and adaptive behavior—provide a foundation for sensing platforms in which detection and information transformation are inherently coupled. Future systems are expected to move beyond architectures composed of discrete components toward integrated material–system frameworks, where sensing, computation, and actuation are co-located and co-designed. In such platforms, materials extend their role beyond signal detection to participate directly in information encoding and pre-processing, enabling structured representations to emerge at or near the point of measurement. This shift reduces reliance on centralized computation and supports low-latency, energy-efficient operation across applications ranging from environmental monitoring and healthcare diagnostics to smart infrastructure and autonomous systems [4,74,75].

A central direction is the development of self-adaptive sensing systems that operate across multiple interacting levels, where material states evolve in response to external stimuli, device characteristics adjust dynamically to local conditions, and system-level models guide decision-making through feedback. The coupling of these processes enables sensing platforms that are not only responsive but context-aware and continuously adaptive, effectively blurring the boundary between sensing and learning [43,52]. These developments also point toward a transformation in how scientific discovery is conducted: in autonomous experimental frameworks, sensing systems can guide exploration through closed-loop operation, linking data acquisition, model inference, and experimental control in real time. Materials with tunable and dynamic responses can therefore function simultaneously as subjects of investigation and as active elements within adaptive workflows, accelerating the identification of new functional materials and emergent phenomena [49,51,74].

Although challenges related to variability, scalability, and integration remain, ongoing progress in non-equilibrium synthesis, heterogeneous integration, and physics-informed machine learning suggests that these limitations are increasingly tractable through coordinated co-design across materials, devices, and algorithms. Looking ahead, the evolution of sensing technologies will be defined not only by improvements in sensitivity and selectivity, but also by the emergence of systems capable of learning, adaptation, and real-time decision-making [74,75,76]. In this paradigm, sensing becomes an active component of intelligent systems that interact dynamically with their environment, marking a transition from passive observation to integrated intelligence. The next generation of sensing platforms will not simply measure physical reality, but will interpret, respond to, and ultimately help guide complex physical systems.

## Data Availability

No new data were created or analyzed in this study. Data sharing is not applicable to this article.
